# Estimation of peer effect in university students’ employment intentions: randomization evidence from China

**DOI:** 10.3389/fpsyg.2023.1241424

**Published:** 2023-12-04

**Authors:** Wenwen Wu, Yongyou Zhong, Guohua Zeng

**Affiliations:** School of Economics and Management, Jiangxi University of Science and Technology, Ganzhou, Jiangxi, China

**Keywords:** employment intentions, peer effect, random class assignment, university students, mechanism

## Abstract

As a key determinant of employment behavior, employment intention is easily affected by the environment, others and their subconsciousness, thus deviating from the optimal decision predicted by the classical economic model. Peers are an important environmental factor that directly affects individual behavior, but their effect on employment intentions has not been fully verified. The paper analyzes the class peer effect on university students’ employment intentions using random class assignment data from a central province in China. It is found that positive peer employment behavior has a significant positive effect on university students’ employment intentions, and this result remains robust after replacing the proxy variables. Further analysis of the peer effect mechanism reveals that the provision and dissemination of school employment information enhances the peer effect in employment intentions, while the help given by parents and family background weakens the peer effect. The results of the dose effect of the peer effect show that the peer effect tends to increase over time.

## Introduction

The world today is experiencing a great change unprecedented in a century, and the global pandemic of the COVID-19 has accelerated the evolution of this great change. Economic globalization has encountered a countercurrent and the development strategy of the international great circle has been obstructed, which has not only had a great impact on China’s macro economy, but also on the employment market of university students. In 2022 the number of graduates in China exceeded 10 million for the first time, and the proportion of unit employment is only about 50%, with up to 18.6% choosing freelance and 15.9% choosing slow employment. In the face of increasingly severe employment situation university students generally adopt a negative attitude and choose “slow employment” to duck out of employment, or even become “lazy employment” (Xia, 2021). Therefore, how to improve the employment intention and promote the employment of university students is of great significance to the realization of personal value, economic development and social stability.

Employment intentions refer to those motivational factors that influence employment behavior and reflect how much effort and time a person is willing to spend in searching for a job (Ajezen, 1991). Due to inertia, bias, and ignorance, individual decisions are often not based on rational thinking and are susceptible to the influence of the environment, others, and their own subconscious, thus deviating from the optimal decisions predicted by classical economic models (Kahneman, 1979; Hansen, 2016; Ju, 2017). Employment decisions are also often influenced by the surrounding environment due to inadequate information and the inability to find employment opportunities. For example, on the one hand, university students’ job search behavior will rely on the help of their peers around them, and on the other hand, they will have a comparative psychology about the job search efforts of their peers, which motivates them to search for jobs. However, the current literature on factors influencing university students’ employment intentions focuses on psychological factors (Fort et al., 2015; Zainal et al., 2020), individual characteristics (Tae-hyun, 2016; Zhang, 2019; Xu, 2022), social capital (Wei, 2009; Lee and Cha, 2014), school type (Song and Yan, 2019; Liu et al., 2021), job market (Li, 2022), and policy environment (Wang and Xianglong, 2021), few studies have considered the influence of surrounding peers on university students’ employment intentions. Even if a few studies have taken into account the influence of peers on university students’ employment intentions, they are mostly qualitative analyses (Zhang and Siqi, 2021), and there is little empirical analysis literature to accurately identify the peer effects existing in university students’ employment intentions.

This paper examines the peer effect of classmates by using “whether or not to participate in internships” and “duration of participation in internships” as the measurement variables of employment intentions. Employment intentions refer to those motivational factors that influence employment behavior and reflect how much effort and time a person is willing to put into job searching. Therefore, employment intention of university students can be measured from the perspectives of “whether there is job searching behavior” and “the degree of effort in job searching.” Considering the possibility of passive job search behavior (during graduation, parents and schools will force graduates to look for jobs), “whether they search for jobs” is replaced by “whether they participate in internships,” and students who participate in internships will have a stronger willingness to work. The duration of internships is used to measure job search efforts, the longer the duration of internships, the harder the students search for jobs, and the stronger their willingness to work. Our empirical results show a significant positive peer effect on university students’ employment intentions from both perspectives, with a 1% increase in the proportion of class peers participating in internships increasing the probability of individual participation by 6.559%. Each 1-unit increase in the average internship time of class peers is associated with a 0.910-unit increase in individual internship time. This result remains robust after replacing the measurement variable of employment intentions. The analysis of the mechanism of influence of the peer effect finds that the provision and distribution of school employment information enhances the peer effect in employment intentions, and the help given by parents and family background weakens the peer effect in employment intentions. Analyzing the heterogeneity of the peer effect results, it is found that undergraduate students and female students receive more peer effect, and there is no significant difference between science and technology students and humanities and social science students. The results of the dose effect of the peer effect show that the peer effect tends to increase over time.

The possible contributions of this paper are, first, to provide evidence of peer effects in the employment behavior of university students. Existing studies on class peer effects have mainly focused on students’ academic performance in China, and although scholars in other countries have noted peer effects in students’ social behavior, they have also mainly focused on the existence of negative externalities in students’ undesirable behaviors, and few studies have analyzed whether there are peer effects in students’ employment behaviors. The second is the analysis using full-sample class data. Most of the international studies on peer effects in individual social behavior are limited to a specific group or randomly selected from the overall class, both of which have problems with small sample sizes, and thus their results are not very persuasive and do not draw general conclusions. This study is based on data from randomly assigned classes across the whole province in China, so the conclusions drawn are more reliable and persuasive. Third, it provides certain policy inspiration for solving the problem of difficult employment of university students. As an important part of talent resources, university students are an important force to promote economic and social development. In recent years, with the continuous development and progress of China’s education level, the number of university students is increasing year by year, and the problems faced by university students’ employment and entrepreneurship situation are gradually highlighted. Facing the increasingly severe employment situation and their own high expectations for their careers, university students are keen on slow employment. Through mechanism analysis, this paper analyzes the influence mechanism of peer effect of university students’ employment intention in terms of help from parents and family background and school employment information provision and release, and the conclusion of this paper has important reference value for how to guide university students’ employment correctly.

## Literature review

### Literature review of university students’ employment intention

The literature on the factors influencing university students’ employment intention mainly focuses on individual, school, family and government. Lots of literature focuses on the individual level and studies the factors influencing employment intentions from the theory of planned behavior. Ajzen (1985) proposes the theory of planned behavior and points out that employment intentions are influenced by attitudes, subjective norms, and perceived behavioral controls. Fort et al. (2015) verifies the association between this theoretical variable and employment intentions in a French sample, and further tests the moderating role of job search experience and two personality dimensions (extraversion and responsibility). In contrast, Lee and Cha (2014) find a mediating role for job search attitude, subjective norms and sense of behavioral control in their study of the effect of the degree of use of corporate job pages on employment intentions. In addition, it is confirmed that the degree of use of corporate job pages has a positive effect on social capital, and the social capital formed has a significant effect on employment intentions.

In the context of rural revitalization strategy, scholars in China are keen to study the factors influencing university students’ rural employment intentions. Xu (2022), from the individual level and the government level, concludes that university students’ rural employment intention is closely related to the gender of individuals, the majors they study, their remuneration packages, and rural policies. Wang and Xianglong (2021) find that the perceived rural employment policy environment has a significant positive effect on the rural employment intentions of university students in agricultural universities. Other scholars have looked at family and school factors, for example, Wei (2009) finds that parents’ social status and social capital have a significant effect on university students’ employment intentions. Liu et al. (2021) find that family characteristics and school type have significant effects on university students’ employment intentions.

Although existing studies have verified the influence of environmental factors on employment intentions, few studies have been conducted on the influence of peers on university students’ employment intentions. In the few literature about the influence of peers on university students’ employment intentions, there are just qualitative analyses, for example, Zhang and Siqi (2021) point out that university students’ entrepreneurial intentions are influenced by their peers around them and there is a strong peer effect, but do not test the role played by peers empirically.

### Literature review of peer effect

Coleman is the first to propose the potential importance of peers in the educational process of students in his 1966 report and finds that in addition to family background, peers have the greatest influence on students’ education. The peer effect, also known as the cohort effect, refers to the externalities of human capital accumulation that result from interactions between people within a group, and the peer effect in education is broadly defined as the influence of the background, behavior, and output of peers within a dormitory, class, grade, or school on student output or behavior (Du and Yuzh, 2016). A large number of studies on peer effects on students’ academic performance have been conducted, but the findings are mixed. Scholars have mostly focused on peer group totality differences at first, studying the effect of average peer academic achievement on students’ academic performance. Most scholars have obtained significantly positive peer effects (McEwan and Soderberg, 2006; Carrell et al., 2009; Ding and Haiping, 2009; Griffith and Rask, 2014; Han, 2022) and some scholars have found no significant effects (Ha, 2016). Since then, some scholars have begun to focus on structural differences in peer groups, i.e., the effect of the degree of class homogeneity on students, and to study the effect of the standard deviation of peer academic performance on students’ academic performance. For example, scholars such as Booij et al. (2016), Cheng (2021), and Yang (2021) find that different degrees of class homogeneity can have different effects on students. And on the base of this, they also find that: the degree of class homogeneity has a strong positive impact on low ability students, and a certain negative impact on high ability students, i.e., the peer effect is heterogeneous. Furthermore, they argue this point from the aspect of gender and socio-economic status of the family. Some scholars have further analyzed the long-term effects of peer effects based on their research. For example, a study of a top 5% comprehensive ranking university in China finds that the academic achievement of roommates has a significant impact on students’ own human capital accumulation, and the peer effect gradually increases over time (Cheng, 2017; Jie et al., 2019), but some scholars also find that the peer effect on academic development gradually weakens over time (Sacerdote, 2001; Ma and Yifan, 2021).

The peer effect includes not only the influence of peers on students’ academic achievement but also on their social behavior, and the closer the outcome variable of peers on students is to social behavior, the greater the spillover effect generated by their peers (Sacerdote, 2011). Some studies have even found that parents have no direct influence on individual behavior and that peers are the only important environmental factor that directly affects individual behavior (Gerstel, 1999). For example, in students’ decisions about whether to join students’ association, one study finds that peers are the main factor in determining whether students join (Marmaros and Sacerdote, 2006). Some scholars have found that peers also have a significant effect on students’ choice of major (Ha, 2016). There is even a significant amount of peer effects for aspects of undesirable peer behaviors, such as internet addiction, delinquency, drug use, alcohol abuse, and pregnancy. For example, Dong and Yuanyuan (2021) and Ning et al. (2021) find that peer effects are a key factor in adolescents’ Internet addiction; peer alcoholism quadrupled the number of students’ alcohol use (Duncan et al., 2005).

In general, the existing studies on peer effects in China mainly focus on students’ academic performance, and although scholars in other countries have noticed peer effects in students’ social behaviors, they also mainly focus on the negative externalities in students’ bad behaviors, and few studies have analyzed whether peer effects exist in students’ employment behaviors and the mechanisms of their existence, and exploring their existence and mechanisms of their effects is beneficial to improving university students’ employment intention and correctly guiding college students’ employment, which has important practical significance. At the same time, most of the studies have been conducted on a specific group or a sample group, and few studies have been conducted to identify the peer effect of the whole sample. This paper draws on the research methods and ideas of the above-mentioned literature to study the marginal effects of class peer effects on the breadth and depth of university students’ employment intentions in China, using a large sample of data from randomly assignment classes.

## Research design

### Data description

Under the Chinese university entrance examination system, students are admitted to the corresponding universities and majors according to their college entrance examination scores and the order of universities and majors which they have applied, and then the admission departments of the universities randomly assign students of the same majors to classes, and then make appropriate fine adjustments to the random assignment results according to the province of origin and gender, i.e., to make the gender ratio and the ratio of places of origin as balanced as possible between classes and avoid students of the same gender or a certain province Concentrated allocation to a certain class.

During normal classes, university students stay in one classroom and different teachers come to the classroom to teach the corresponding subjects. Moreover, Chinese universities put great emphasis on collection construction of class, and students in a class are often organized together for team activities. Therefore, university students in the same class have intensive interactions in terms of employment purposes.

This paper uses the employment survey data of university graduates in a central province in China in 2018, which was organized and implemented by the employment office of the provincial education department and included basic demographics of graduates, class and school information, and internships, the number of resumes placed, the number of job fairs attended, the number of interviews attended, and the majors of minors. Students’ employment intentions were measured by their internships. Demographic characteristics include gender, ethnicity, place of origin, family economic background, and parental education level. The data covers all classes in all colleges and universities in the whole province, including 169,037 graduates in 3,746 classes in 60 colleges and universities, and the valid sample size is 99,132 after removing missing values of classes. Since each student in our sample is randomly assigned to a classroom, it alleviates our concerns about students self-selecting classrooms. Table 1 reports summary statistics for our main outcome and control variables.

**Table 1:** Summary statistics for variables.

Variables	Obs	Mean	Sd	Min	Max
Dependent variable					
Internship time	99,132	3.954	2.312	0	7
Whether to do internship	99,132	0.927	0.260	0	1
Core independent variable					
Average internship time of classmates	99,132	3.952	1.404	0.400	7
Proportion of classmates participating in internship	99,132	0.926	0.0824	0.300	1
Individual and family characteristics					
Male	99,132	0.489	0.500	0	1
Urban	99,132	0.113	0.316	0	1
Minority	99,132	0.0366	0.188	0	1
Party membership	99,132	0.0806	0.272	0	1
Poor	99,132	0.147	0.354	0	1
Father’ s education	99,132	1.415	0.844	0	5
Father’ s job	99,132	0.0827	0.275	0	1
Mother’ s job	99,132	0.00516	0.0717	0	1
Average individual and family characteristic variables of classmates					
Proportion of male	99,132	0.489	0.329	0	1
Proportion of urban	99,132	0.112	0.0883	0	0.714
Proportion of minority	99,132	0.0366	0.0882	0	1
Proportion of Party membership	99,132	0.0805	0.0972	0	1
Proportion of poor	99,132	0.147	0.160	0	1
Father’ s average education	99,132	1.415	0.259	0.600	3.111
Father’ s average job	99,132	0.0827	0.0719	0	0.667
Mother’ s average job	99,132	0.00516	0.0148	0	0.133
Class scale	99,132	31.97	15.61	10	163
Mechanism variable					
Provision and dissemination of school employment information	99,132	3.719	0.959	1	5
Help from parents and family background	99,132	0.280	0.449	0	1

### Variable measures and explanations

#### Outcome variables

University students’ employment intentions. The degree of university students’ employment intention is measured by “internship time” and “whether to do internship.” The value of “internship time” is 0–7, and the larger the value of the variable is, the longer the internship time is, the more efforts university students have made for searching for job, and the stronger their employment intentions are. “Whether to do internship” is a dummy variable, if the student participates in the internship, the value is 1, otherwise the value is 0.

#### Core explanatory variables

Peer variables. The employment intention of peers is expressed as (employment behavior of the whole class - employment behavior of the ith student)/(class size – 1); i.e., average time of internship by peers is equal to (sum of internship time of the whole class – internship time of the ith student)/(class size of the ith student – 1); percentage of peer participation in internships is equal to (number of students participating in internship of the whole class - whether the ith student participates in internship)/(class size of the ith student – 1). The specific formula is as follows.


(1) $$\mathrm{Pee}r_{i_{c},gs}=\frac{1}{N-1}\overset{N}{\underset{\begin{array}{c} J=1\\ J\neq i \end{array}}{\sum }}y_{j}$$

where N represents the total number of students in class c of major g at university s, and y_j_ represents a continuous variable for the internship time of student i’s peer j in class c as well as a dummy variable for whether to do internship.

#### Moderating variables

The moderating variables include “Help from parents and family background” and “Provision and dissemination of school employment information.” “Help from parents and family background” is a dummy variable with a value of 1 if parents and family background help, otherwise it is 0. “Provision and dissemination of school employment information “is a continuous variable with five levels from 1 to 5. The larger the value is, the more thoughtful and comprehensive services universities provide.

#### Control variables

The control variables mainly include personal characteristics, family background, class characteristics, university, major and other variables that may affect university students’ employment intention.

### Research methodology

To investigate how peers’ employment behavior affects students’ employment intentions, we implemented a linear mean model that has been widely used in the literature (Jonathan et al., 2009; Lu and Anderson, 2015). We used the following regression model.


(2) $$\begin{array}{l} Y_{i_{c},gs}=\beta_{0}+\beta_{1}\mathrm{Pee}r_{i_{c},gs}+\beta_{2}X_{i_{c},gs}+\beta_{3}X\bar{{}_{i_{c},gs}^{CM}}\\ \;+\beta_{4}P_{i_{c},gs}+\alpha_{gs}+\mu_{i_{c},gs} \end{array}$$

Ajezen (1991) in the theory of planned behavior points that the immediate prerequisite for any behavior is the intention to perform that behavior, and the determinants that influence intention include attitudes, subjective norms, and perceived behavioral control. Subjective norm is a social factor that refers to the perceived social pressure which requires people do something or not. It reflects the influence of significant others or groups on individual behavioral decisions. Therefore, peers have an effect on university students’ employment intention through subjective norms.

The peer effect occurs mainly through knowledge spillover and social pressure (Cornelissen et al., 2017). On the one hand, the mutual transfer of employment information among classmates reduces job search costs and makes job search easier; on the other hand, the positive employment behavior of peers generates some social pressure on university students and motivates individuals to increase their job search efforts. Based on this, this paper studies the peer effect of university students’ employment behavior from two perspectives: whether to search for a job and the degree of job search effort, and defines the proportion of job search as the breadth marginal effect of the peer effect and the degree of job search effort as the deep marginal effect of the peer effect. Considering that there may be passive job search behavior, whether university students participate in internship during school is substituted for whether they search for jobs, and university students who participate in internship will have stronger employment intention. As for the degree of job search effort, it can be measured by the internship time. The longer the internship time is, the more efforts they have made for searching for job, and the stronger their employment intentions are.

From the breadth marginal effect perspective the explanatory variable $Y_{i_{c},gs}$ denotes a dummy variable for whether student i in class c of major g at university s participates in internship, taking a value of 1 if the student participates in internship and 0 otherwise. at this point, the peer variable indicates the proportion of peers participating in the internship except student i in class c of major g at university s.

From the depth marginal effect perspective, the explanatory variable $Y_{i_{c},gs}$ denotes a continuous variable for internship time that student i in class c of major g at university s participates in.

$X_{i_{c},gs}$ are individual and family characteristic variables of university students. $X_{i_{c},gs}^{\vec{CM}}$ Represents the average individual and family characteristic variables of classmates.

$P_{i_{c},gs}$ are class characteristics variables; $\alpha_{gs}$ denotes university and major fixed effects and $\mu_{i_{c,}gs}$ is a random disturbance term.

The coefficient we are concerned with is β_1_, which reflects the effect of peer employment behavior on university students’ employment intentions.

Manski (1993) points out the reflection problem, i.e., the identification of peer effects, that arises when a researcher tries to infer whether the average behavior of a group influences the behavior of the individuals that comprise the group. Generally speaking, the common observation that individuals belonging to the same group tend to behave similarly contains three types of effects: (a) endogenous effects, wherein the propensity of an individual to behave in some way varies with the behavior of the group; (b)exogenous effects, wherein the propensity of an individual to behave in some way varies with the exogenous characteristics of the group; (c)correlation effects, wherein individuals in the same group tend to behave similarly including relevant group factors, i.e., because they have similar individual characteristics (e.g., students with good grades form a class) and common environment factors, i.e., facing similar institutional environments (e.g., students face the same teacher).

To address this identification concern, this paper first uses random class assignment data to remove relevant group factors of correlation effects. Since random class assignment is conducted within schools of the universities, the choice of university and major may not be random, so university-major fixed effects α_cs_ are included in the regressions to control for all university- and major-level factors that may influence students’ university and major choice decisions. In addition, the common environment factors of correlation effects are eliminated by further controlling the class characteristics. What’s more, by controlling for average individual and family characteristics for classmates, we can exclude exogenous effects, which led to unbiased estimates of β_1_.

### Randomness test for class assignment

In order to verify the reliability of random assignment, we conducted a test. Using the random test method of Sacerdote (2001) for reference, if the hypothesis of random class assignment in schools is established, the characteristic variables of individual students have nothing to do with the average characteristic variables of classmates after controlling the fixed effect of school and major.


(3) $$X_{igs}=\alpha +\beta X\begin{array}{c} \bar{CM}\\ igs \end{array}+\alpha_{gs}+\mu_{igs}$$

As shown in the model, represents the characteristic variables, including whether they are ethnic minorities, whether they were born in urban areas, whether they are party members, whether they are male, and their parents’ educational level and occupations, $X\begin{array}{c} \bar{CM}\\ igs \end{array}$ represents the average characteristics of classmates in the class of student i, including the proportion of ethnic minorities, urban students and party member and so on. Because the class assignment is carried out within the college of the university, the model further adds the fixed effect of school and major. Β is the coefficient we are concerned about. If there is no significant difference between β and 0, it means that there is no correlation between students’ characteristics in the process of class allocation, and classes are randomly allocated.

Table 2 lists the results of random test, and it can be found that individual characteristics are not related to peer characteristics in most variables, indicating that the overall randomness of class allocation is good.

**Table 2:** Random test results of class assignment.

Classmates mean characteristics	Dependent variable								
	Ethnicity	City-born	Party membership	Gender	Poor	Father’s education	Mother’s education	Father’s job	Mother’s job
Ethnicity	0.845***								
	(0.00628)								
City-born		0.00522							
		(0.0113)							
Party membership			0.00696						
			(0.00886)						
Gender				0.00944					
				(0.03830)					
Poor					0.00831				
					(0.06600)				
Father’s education						0.00574			
						(0.0104)			
Mother’s education							0.00430		
							(0.0315)		
Father’s job								0.00363	
								(0.0127)	
Mother’s job									0.00750
									(0.0155)
Observations	99,132	99,132	99,132	99,132	99,132	99,132	16,656	99,132	99,132
*R*-squared	0.160	0.022	0.066	0.387	0.141	0.034	0.025	0.014	0.001

Own characteristics are regressed on classmates’ average characteristics with school and major fixed effects. ***, **, * denote 1, 5, 10% significant levels, respectively. All regressions are OLS and standard errors are adjusted for heteroscedasticity.

## Empirical analysis

### Empirical analysis of the impact of positive peer employment behavior on university students’ employment intentions

Table 3 shows the estimated results of the peer effect on university students’ employment intentions. Columns (1) and (2) report the OLS regression results of the peer effect on university students’ internship time, and columns (3) and (4) report the Logit regression results of the peer effect on the percentage of participation in internship. Overall, peer’s active employment behavior has a significant role in promoting university students’ employment intentions. With the increase of peer internship time, the internship time of university students will also increase significantly; The higher the proportion of peers participating in internships, the higher the probability of university students’ internships.

**Table 3:** Baseline regression results.

	(1)	(2)	(3)	(4)
Variable	Internship time		Whether to do internship	
Core independent variable				
Peer	0.912***	0.910***	6.692***	6.559***
	(0.00402)	(0.00404)	(0.117)	(0.119)
constant	0.193***	0.333***	−3.762***	−3.032***
	(0.0305)	(0.0527)	(0.121)	(0.156)
Control variable				
Individual and family characteristic variables	Yes	Yes	Yes	Yes
Average individual and family characteristic variables of classmates	No	Yes	No	Yes
Class variable	Yes	Yes	Yes	Yes
School and major fixed effects	Yes	Yes	Yes	Yes
observations	99,132	99,132	99,132	99,132
*R*^2^/pseudo *R*^2^	0.319	0.319	0.0595	0.0621

In column (1) and column (2), each cell represents a separate regression, in which the independent variable is graduates’ internship time. The regressions are OLS. And in column (3) and column (4) the independent variable is whether graduates do internship. The regressions are Logistics. specifications in column (2) will be more strict than column (1), and specifications in column (4) will be more strict than column (3). Standard errors are adjusted for heteroscedasticity. ***, **, * denote 1, 5, 10% significant levels, respectively.

Specifically, considering only student characteristics, family background characteristics, class characteristics and university and major fixed effects, a 1-unit increase in peer internship time significantly increases university students’ internship time by 0.912 units; a 1% increase in the proportion of peer participation in internship increases the probability of university students’ internship by 6.692%, with all statistical tests of the parameters reaching the 1% significance level. After further controlling for other variables that may affect the outcome variables, a 1-unit increase in peer internship time significantly increases university students’ internship time by 0.910 units; a 1% increase in the proportion of peers participating in internships increases the probability of university students’ internships by 6.559%, and the parametric statistical tests still reach the 1% level of significance. Therefore, the positive employment behavior of peers has a significant promotion effect on university students’ employment intentions.

### Mechanism analysis of the influence of peer positive employment behavior on university students’ employment intention

In this part, the mechanism of the peer effect of university students’ employment intention is studied at the family level and school level through the help from parents and family background and provision and dissemination of school employment information. (a) At the family level, parents give help to their children in the process of job search, which often directly affects their employment intentions (Wei, 2009). Students with good family economy have little employment pressure, so they do not pay attention to the exchange of employment information with their peers, which will weaken the help from class peers to individuals in the process of job search, thus weakening the peer effect. (b) At school level, the service about provision and dissemination of employment information provided by university can not only directly reduce the job search cost and provide a broad platform and channel for university students to search for jobs, but also help create an employment atmosphere, enhance the multiplier effect of peer effect among classmates, and promote university students’ willingness to search for job. In general, the provision and dissemination of school employment information can, on the one hand, improve university students’ employment intention and have a substitute effect on the peer effect; on the other hand, it can create an employment atmosphere and enhance the peer effect. Therefore, the influence mechanism of peer effect is estimated by multiplying these mechanism variables with peer variables, respectively, in the form of interaction terms.

As shown in Table 4, columns (1) and (3) report the moderating effect of school employment information provision and dissemination on the peer effect, while columns (2) and (4) report the moderating effect of help from parent and family background on the peer effect. Overall, the coefficient of school employment information provision and dissemination is significantly positive, and the coefficient of its interaction term with the peer variable is also significantly positive, indicating that school employment information provision and dissemination not only directly increases university students’ employment intentions, but also indirectly increases university students’ employment intentions by enhancing the peer effect. Specifically, for each unit increase in the provision and dissemination of school employment information, the peer effect on university students’ internship time will increase by 0.01 units, and the statistical test of the parameter will reach the 5% level of significance; the peer effect on university students’ internship probability will increase by 0.387 units, and the statistical test of the parameter will reach the 1% level of significance.

**Table 4:** Results of the mechanism analysis of the peer effect.

Outcome variable	Internship time		Whether to do internship	
	(1)	(2)	(3)	(4)
Mechanism variables *X*	Provision and dissemination of school employment information	Help from parents and family background	Provision and dissemination of school employment information	Help from parents and family background
X × Peer	0.010**	−0.034***	0.387***	0.067
	(0.004)	(0.008)	(0.117)	(0.258)
X	0.042**	0.156***	−0.135	0.079
	(0.018)	(0.036)	(0.104)	(0.230)
Peer	0.889***	0.934***	5.282***	6.691***
	(0.016)	(0.004)	(0.434)	(0.136)
Observations	99,132	99,132	99,132	99,132
*R*^2^/pseudo *R*^2^	0.320	0.318	0.064	0.059
Individual and family characteristic variables	Yes	Yes	Yes	Yes
Average individual and family characteristic variables of classmates	Yes	Yes	Yes	Yes
Class variable	Yes	Yes	Yes	Yes
School and major fixed effects	Yes	Yes	Yes	Yes

Robust standard errors in parentheses. ***, **, * denote 1, 5, 10% significant levels, respectively.

It can be found that the coefficient of help from parents and family background is significantly positive, but the coefficient of its interaction term with the peer variable is significantly negative, that is, the help from parents and family background to their children in the job search process directly enhances university students’ willingness to search for a job, but weakens the influence of peers on them. Specifically, the peer effect on university students’ internship time decreases by 0.034 units for each unit of parental and family background help, and the statistical test of the parameter reaches 1% significance level; although the peer effect on university students’ internship probability increases by 0.067 units, it is not statistically significant.

### Heterogeneity analysis of the effect of positive peer employment behavior on university students’ employment intentions

This part discusses the heterogeneous influence of peer effect of university students’ employment intention from three aspects: school level, major and gender. Song (2012) points out that students of different genders and different disciplines have different employment awareness and different sensitivity to peer employment behavior, and thus may be affected by peer effects differently. Liu et al. (2021) finds that school type has a significant influence on students’ employment intentions. The better the university the university students attend, the stronger the employment atmosphere, and consequently the higher the students’ employment intentions and the greater the influence from their peers. This section uses subsample regressions to estimate the heterogeneity of peer effects.

As shown in Table 5, overall, we find evidence that peer effect on university students’ employment intentions is stronger for undergraduate students and female students. The possible reason is that the employability of undergraduates is more outstanding than junior college students, so there are more enterprises to choose from for undergraduates, and the employment intention will be stronger; there is no significant difference between science and liberal arts students. The students majored in science are influenced by their peers around them and focus on extending their internship time. The students majored in liberal arts are influenced by their peers and focus on increasing the probability of their own successful internships.

**Table 5:** Heterogeneity analysis results of peer effect.

Outcome variable	Internship time		Whether to do internship	
	Junior college students	Undergraduate	Junior college students	Undergraduate
Peer	0.827***	0.943***	6.082***	6.986***
	(0.011)	(0.004)	(0.182)	(0.157)
Observations	34,981	64,151	34,981	64,151
*R*^2^//pseudo *R*^2^	0.146	0.392	0.053	0.061
	Science	Liberal arts	Science	Liberal arts
Peer	0.947***	0.879***	6.475***	6.790***
	(0.005)	(0.007)	(0.189)	(0.151)
Observations	45,544	53,588	45,544	53,588
*R*^2^/pseudo *R*^2^	0.416	0.208	0.054	0.062
	Female	Male	Female	Male
Peer	0.951***	0.888***	7.858***	5.622***
	(0.005)	(0.006)	(0.167)	(0.164)
Observations	50,675	48,457	50,675	48,457
*R*^2^/pseudo *R*^2^	0.369	0.268	0.080	0.042
Individual and family characteristic variables	Yes	Yes	Yes	Yes
Average individual and family characteristic variables of classmates	Yes	Yes	Yes	Yes
Class variable	Yes	Yes	Yes	Yes
School and major fixed effects	Yes	Yes	Yes	Yes

Robust standard errors in parentheses. ***, **, * denote 1, 5, 10% significant levels, respectively.

Specifically, for the peer effect in the presence of internship time, undergraduate students receive 0.116 units more than junior college students; female students receive 0.063 units more than male students; students majored in science receive 0.068 units more peer effect than those who are majored in liberal arts; for the peer effect in the whether to do internship, undergraduate students receive 0.904 units more than junior college students, and Female students receive 2.236 units more than male students, however, students majored in science receive 0.315 units less peer effect than those who are majored in liberal arts.

### Dose effects of peer effects

This part examines the dosage effect of the peer effect on university students’ employment intention through the difference length of schooling. On the one hand, the longer the time spent in school, the more frequent, fuller communication and closer relationship with classmates, and the greater the peer effect you will receive (Dahl et al., 2014). On the other hand, as time goes by, the interaction will not be limited to classmates, but will seek like-minded people according to their own interests and hobbies, and will gradually expand to include old folks, members in the student associations they join, etc., and the class peer effect will gradually diminish. Based on the baseline model, the dose-effect model adds the school length variable and its interaction with peer variables.

From Table 6, it can be concluded that the peer effect tends to increase over time. For each additional year of time spent with peers, the peer effect received by university students increased by 0.004 units in internship time and 0.228 units in internship probability. Using the two-year educational system as a reference, it can be found that the peer effect received by both three-year and four-year university students is decreasing, but the peer effect received by the five-year system is increasing and the increase is greater than the decrease in the three-year and four-year systems, making the final result show a positive effect. The possible reason is that compared with two-year graduate students, three-year junior college students and four-year undergraduate students pay less attention to the interpersonal relationship with their classmates and receive less peer effect. Most of the five-year students major in medicine and will have a large number of courses to study due to the specificity and importance of the profession, so they do not have much spare time to communicate with peers outside the class, and it is difficult for other peers to have a strong substitution effect on class peers, resulting in a large class peer effect.

**Table 6:** Analysis results of dose influence on peer effect.

	(1)	(2)	(3)	(4)
Variable	Internship time		Whether to do internship	
Peer	0.909***	0.927***	5.755***	6.720***
	(107.47)	(70.48)	(37.50)	(34.37)
Peer × Two years				
(baseline)				
Peer × Three years		−0.021*		−0.332**
		(−1.68)		(−2.11)
Peer × Four years		−0.047***		−0.180
		(−3.70)		(−1.14)
Peer × Five years		0.004		0.421**
		(0.28)		(2.48)
Peer × school length	0.004**		0.228***	
(continuous variable)	(2.27)		(9.71)	
				
Observations	99,132	99,132	99,132	99,132
*R*^2^/pseudo *R*^2^	0.318	0.319	0.061	0.062
Individual and family characteristic variables	Yes	Yes	Yes	Yes
Average individual and family characteristic variables of classmates	Yes	Yes	Yes	Yes
Class variable	Yes	Yes	Yes	Yes
School and major fixed effects	Yes	Yes	Yes	Yes

T-statistics in parentheses. ***, **, * denote 1, 5, 10% significant levels, respectively.

### Robustness test

This section tests the robustness of the results by displacing the measurement variables of employment intentions. The number of resumes submitted, the number of job fairs attended and the number of interviews attended by university students, and whether they minor in other majors are used to measure the strength of employment intentions.

From Table 7, it can be concluded that the coefficients of the peer variables are significantly positive whether it is the number of resumes submitted, the number of job fairs attended, the number of interviews attended or the participation in minors, and the statistical tests of the parameters all reach the 1% level of significance, that is, they verify the conclusion of this paper that there is a significant positive peer effect on university students’ employment intention.

**Table 7:** Regression results of displacement outcome variables.

	(1)	(2)	(3)	(4)
Variable	Number of resumes submitted	Number of job fairs attended	Number of interviews attended	Minor or not
Peer	0.455***	0.442***	0.543***	5.422***
	(0.0129)	(0.0114)	(0.00983)	(0.0817)
Constant	0.976***	1.061***	1.294***	−3.203***
	(0.0265)	(0.0265)	(0.0340)	(0.0900)
Observations	64,004	64,004	64,004	99,132
*R*^2^/pseudo *R*^2^	0.043	0.056	0.070	0.083
Individual and family characteristic variables	Yes	Yes	Yes	Yes
Average individual and family characteristic variables of classmates	Yes	Yes	Yes	Yes
Class variable	Yes	Yes	Yes	Yes
School and major fixed effects	Yes	Yes	Yes	Yes

Robust standard errors in parentheses. ***, **, * denote 1, 5, 10% significant levels, respectively.

## Conclusion

Employment intention, as a key determinant of employment behavior, is easily influenced by the environment, others and one’s own subconscious, thus deviating from the optimal decision predicted by classical economic models. Peers are important environmental factors that directly influence individual behavior, and their effects on employment intentions have not been fully verified. In this paper, we analyze the class peer effect on university students’ employment intentions using random class assignment data from a central province in China.

The results of the empirical analysis show that (a) Peer’s active employment behavior has a significant positive effect on university students’ employment intention. Specifically, when the proportion of class peers participating in internship increases by 1%, the probability of individual participation in internship increases by 6.559%; for each additional unit of average internship time of class peers, the internship time of individual increases by 0.910 units. (b) Further analysis of the mechanism of peer effect reveals that the provision and dissemination of school employment information has a significant positive impact on peer effect, while the help from parents and family background has a significant negative effect on peer effect. (c) Analyzing the heterogeneity of the peer effect results, it is found that undergraduate students and female students receive more peer effect, and there is no significant difference between science and liberal arts among students. (d) The analysis of the dose effect of peer effect finds that the peer effect tends to increase over time.

Through the conclusion, it can be concluded that there is a significant peer effect in employment intention. How to correctly guide the employment of university students, in addition to pay attention to the factors of university students themselves, the impact of the environment on the individual cannot be ignored. As an important environmental factor, we should make full use of the influence of peers on individuals to improve university students’ employment intention and properly guide them to employment.

Based on this, the following suggestions are put forward.

(1) Establish the power of role models to lead the way forward. This paper concludes that the active employment behavior of peers can promote university students’ employment intention. Therefore, it is important for us to pay attention to our peers who are active in employment and to develop our own belief in employment.

(2) Strengthen school employment services. School employment service can not only directly promote the overall employment behavior of university students, but also enhance the peer effect of employment intention and indirectly promote the employment intention of university students.

(3) Strengthening parental guidance on students’ employment awareness. This paper concludes that the help from parents and family background will weaken the peer effect of university students’ employment intention. The possible reason is that university students are emboldened by the advantage of family background and often do not attach importance to employment, so parents should guide university students’ employment awareness correctly.

This study also has some limitations. Due to the availability of data, this paper examines the peer effect in employment intention from the class level, and does not further focus on the influence of peers on individual employment intention from the dormitory level; in addition, the influence mechanism of the peer effect is explored from only two perspectives of the family and the school, and there may be other aspects, which are also worthy of research in the future.

## Data availability statement

The data analyzed in this study is subject to the following licenses/restrictions: the data that support the findings of this study are available on request from the corresponding author. The data are not publicly available due to them containing information that could compromise research participant privacy/consent. Requests to access these datasets should be directed to GZ, zensoyou@163.com.

## Ethics statement

Ethical review and approval was not required for the study on human participants in accordance with the local legislation and institutional requirements. Written informed consent from the participants was not required to participate in this study in accordance with the national legislation and the institutional requirements.

## Author contributions

GZ was responsible for the writing, conception and design of the article, and data collection. YZ and WW provided critical review of the intellectual content of the article and corresponding work support. All authors contributed to the article and approved the submitted version.
